# Spontaneous Pregnancy After the Removal of Long-Term Retained Laminaria

**DOI:** 10.7759/cureus.54278

**Published:** 2024-02-16

**Authors:** Mika Miyamori, Akihiro Hamuro, Kohei Kitada, Takuya Misugi, Daisuke Tachibana

**Affiliations:** 1 Obstetrics and Gynecology, Osaka Metropolitan University Graduate School of Medicine, Osaka, JPN

**Keywords:** laparoscope, hysteroscope, iatrogenic complication, laminaria tents, osmotic cervical dilator

## Abstract

We report the case of a woman with laminaria retention up to six years, followed by spontaneous pregnancy after the removal by hysteroscope of the intrauterine retained laminaria. A 26-year-old woman (G1P0) visited our hospital with complaints of prolonged menstrual bleeding, dyspareunia, and infertility. She had a history of dilatation and evacuation (D&E) at nine weeks of gestation six years earlier. A transvaginal ultrasound showed an artifact, and hysteroscopy revealed a long foreign body, which was suspected to be a laminaria retained after the prior abortion. In the hysteroscopic surgical procedure, the laminaria was cut, and the two halves were excised using resectoscope electrodes and hooked to the electrodes for removal. Thereafter, a year later, she conceived spontaneously and gave birth to a baby by cesarean delivery due to the arrest of labor progress. We are the first to present a pregnant case after the removal of a six-year retained laminaria.

## Introduction

Osmotic cervical dilators (e.g., laminaria tents) have been widely used in preparation prior to dilatation and evacuation (D&E) [1]. Although dilator complications, such as impaction, displacement, and infection, have been reported, the long-term retention of laminaria tents seems to be quite rare [1]. Undetected long-term laminaria retention may cause complications such as menstrual abnormalities, dysmenorrhea, endometritis, and infertility [2,3]. When fragments of laminaria remain in the uterine cavity, it is very difficult to detect and remove them due to their small size and their severe adhesion with scarred endometrium [3,4]. Moreover, there are reports of even legal issues due to laminaria retained [1]. It is important to avoid complications with the correct use of laminaria. Herein, we first report a case of a woman with laminaria retention up to six years, followed by spontaneous pregnancy after the removal of the intrauterine retained laminaria.

## Case presentation

**Figure 1:**
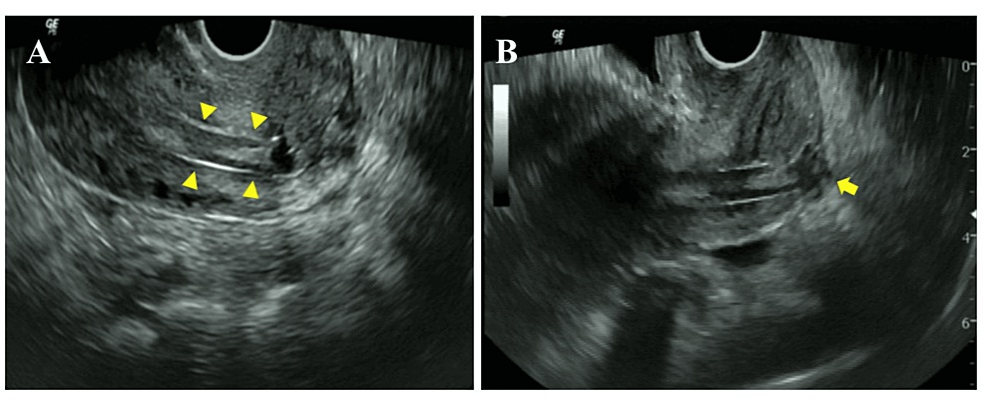
Transvaginal ultrasound images A: Image of artifact in the uterine cavity (arrowheads indicate an artifact in the uterine cavity); B: Image of partial intrusion to the posterior myometrium of the uterus (the arrow indicates a finding suspicious of partial intrusion to the posterior myometrium of the uterus).

A 26-year-old woman (G1P0) visited our hospital with complaints of prolonged menstrual bleeding, dyspareunia, and infertility. She had a history of dilatation and evacuation (D&E) at nine weeks of gestation six years earlier. A transvaginal ultrasound showed a 1 x 5 cm sized artifact which was found to be partially intruding into the myometrium (Figures 1A, 1B), although there were no abnormal findings at the vaginal inspection.

**Figure 2:**
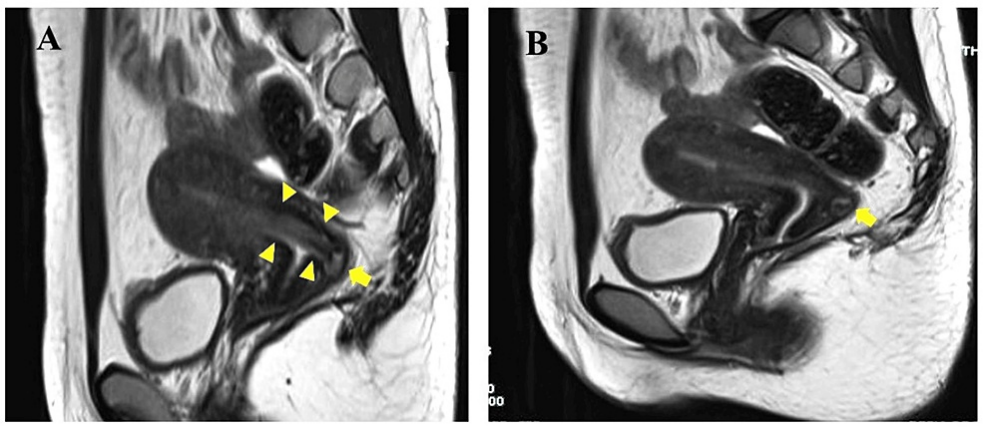
MRI T2-weighted sagittal section of the uterus A: A sticklike image with an iso-intensity in T2-weighted images (arrowheads indicate an artifact in the uterine cavity, arrows indicate a finding suspicious of partial intrusion to the posterior myometrium of the uterus); B: Suspicious finding of partial intrusion to the posterior myometrium (the arrow indicates a finding suspicious of partial intrusion).

Magnetic resonance imaging (MRI) also showed a sticklike object with a low intensity in T2-weighted images (Figures 2A, 2B).

**Figure 3:**
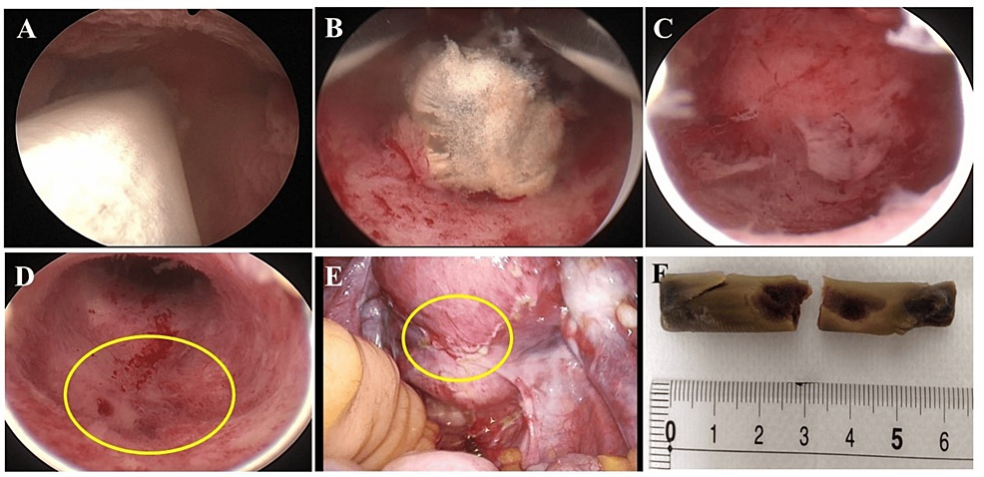
Findings of surgery and removed laminaria A: Hysteroscopy revealed a long foreign body suspected to be a retained laminaria; B: Hysteroscopic surgery (the laminaria was excised in two halves using resectoscope electrodes); C: Hysteroscopic surgery (the endometrium was intact after removal); D: Hysteroscopic surgery (the mark of the circle is an area suspected of laminaria intrusion); E: Laparoscopic surgery (the mark of circle is an area suspected of laminaria intrusion). There was no visual finding of intrusion into the myometrium; F: The removed object was 6 cm long and was visually identified as a laminaria.

Further examination by hysteroscopy revealed a long foreign body, which was suspected to be a laminaria retained after the prior abortion (Figure 3A). Considering the risk of uterine injury, such as perforation, the foreign body was removed using a hysteroscope and with careful observation under a laparoscope. In the hysteroscopic procedure, the laminaria was cut, and the two halves were excised using resectoscope electrodes and hooked to the electrodes for removal (Figures 3B, 3C). The posterior myometrium of the lower uterus, which was thought to be partially intruded into the myometrium, showed no abnormal findings under laparoscopy (Figures 3D, 3E). The excised 6 cm long laminaria is shown in Figure 3F. As the endometrium was intact after removal, we didn't place any intrauterine device to prevent adhesions in the uterine cavity. Thereafter, the patient’s prolonged menstrual bleeding and dyspareunia gradually improved during the postoperative course, and a year later she conceived spontaneously and gave birth to a healthy baby (4,020g) by cesarean delivery due to the arrest of labor progress. The placenta was smoothly separated from the uterine wall, and there was no adhesion around the uterus or adnexa, including the Douglas’ pouch. She was discharged from the hospital with a good post-delivery course, and no problems were observed thereafter.

## Discussion

Osmotic dilators have long been used to mature and soften the cervix for obstetric and gynecologic procedures. Softening and dilating the cervix decreases the risk of cervical injury and uterine perforation and increases the safety of intrauterine manipulation [1]. Laminaria manufactured from a type of seaweed kelp (laminaria japonica) were first used in the United States over 100 years ago and continue to be a useful instrument to date [5]. However, problems caused by laminaria have also been reported. Laminaria becomes larger with moisture and dumbbell-like in the uterine cervix, which can cause resistance to removal. The laminaria may then break off and remain in the uterine cavity [2-4]. When fragments of laminaria remain in the uterine cavity, it is very difficult to detect and remove them due to their small size and their severe adhesion to scarred endometrium [4]. Furthermore, intrauterine laminaria are thought to be difficult to detect via gynecological examinations such as ultrasound imaging [6,7].

Complications such as menstrual abnormalities and infertility caused by undetected laminaria retention could lead to serious mental problems and even legal issues [1]. In order to reduce the risk of being involved in litigation, it is necessary to follow the product documentation for laminaria use precisely as well as the informed written consent before the procedure. According to the product document for Laminaria japonica, the steps of procedures are required [8]. Among the several steps, the important points are to insert two or more laminaria with forceps to a depth slightly beyond the internal cervical os (not too deep) and to confirm the number of removed laminaria and any damage to them. We obstetricians and gynecologists should keep these things in mind when using laminaria.

## Conclusions

We were the first to present a pregnant case after the removal of a six-year retained laminaria. Although retained laminaria can cause serious complications if unnoticed, our report suggests that spontaneous pregnancy and even successful delivery may be anticipated after their removal. In order to reduce the risk of complications and litigations, it is especially necessary to confirm the number of removed laminaria and any damage to them and to get informed written consent before the procedure. We hope that our report will encourage women in distress by D&E and help obstetricians and gynecologists practice proper medical care.
